# A Mixed-Methods Study of Women’s Empowerment through Physical Activities: Relationships with Self-Efficacy and Physical Activity Levels

**DOI:** 10.3390/jfmk8030118

**Published:** 2023-08-12

**Authors:** Aspen E. Streetman, Madiera M. Lister, Averie Brown, Halle N. Brin, Katie M. Heinrich

**Affiliations:** Department of Kinesiology, Kansas State University, Manhattan, KS 66506, USA; mmlister@ksu.edu (M.M.L.); averiebrown@ksu.edu (A.B.); hbrin@ksu.edu (H.N.B.)

**Keywords:** exercise, resistance training, cardiorespiratory training, gym access, women’s health

## Abstract

Participation in empowering physical activities may increase self-efficacy and facilitate long-term engagement. This explanatory sequential mixed-methods study examined the relationship between physical activity empowerment, exercise self-efficacy, and engagement. Midwestern women (*N* = 147) aged 18–65, 90% white, completed an online cross-sectional survey that captured exercise engagement and self-efficacy for exercise. Participants entered up to five types of physical activities and ranked them from most to least empowering. Physical activities were coded by training type for statistical comparisons using independent t-tests. After survey completion, seventeen women completed a 30 min, 8-question semi-structured interview. Women ranked resistance training as the most empowering physical activity type (38%), followed by running (14%). Total and moderate-to-vigorous physical activity and self-efficacy for exercise scores did not vary between women empowered by cardiorespiratory or resistance training (i.e., total physical activity *t*(136) = 1.13, *p* = 0.11; moderate-to-vigorous physical activity *t*(136) = 2.42, *p* = 0.06; and self-efficacy for exercise *t*(136) = 0.66, *p* = 0.07). Themes identified from the interviews included: (1) women’s physical activity participation barriers are gender-centric, (2) physical activity participation benefits extend beyond physical health, (3) some exercise types are more empowering than others, and (4) empowerment and enjoyment are closely related. Exploring empowerment in exercise may reveal mechanisms to facilitate exercise self-efficacy and engagement in physical activity.

## 1. Introduction

Increasing women’s empowerment and facilitating gender equality are critical global priorities. The United Nations General Assembly’s top five sustainable development goals include: “Goal 5: Achieve gender equality and empower all women and girls” [1]. According to empowerment theory, empowerment is a multidimensional construct that involves an individual, their friends and family, and the community in which they live [2,3]. Individual empowerment describes how people gain autonomy and control [2,3]. Empowerment theory dictates that empowerment varies between individuals as the value orientation of empowerment is influenced by individual goals, aims, and strategies for implementing change [2]. Thus, empowerment theory serves as a model explaining the processes and outcomes of empowerment. For example, supportive friends and family may aid in the empowerment process, while an empowerment theory outcome could be having power and control over personal circumstances in the face of obstacles [2,3]. Moreover, previous research suggests that feelings of empowerment in one setting may transfer to another [1,4]. Therefore, fostering and promoting empowerment in exercise may be a powerful mechanism to increase women’s empowerment in other areas of life. 

Increasing empowerment among women through physical activity is critical since women are less likely than men to meet the physical activity guidelines. Globally, 17.4% of women and 23.5% of men meet the guidelines. In the United States, the gender gap is more pronounced (i.e., 20.9% of women vs. 27.2% of men) [5,6,7]. The World Health Organization guidelines exactly mirror those of the US Department of Health and Human Services for adults and include at least 150 min of moderate-intensity aerobic activity, or 75 min of vigorous-intensity aerobic activity, or an equivalent combination of both, and at least two days of muscle-strengthening activity involving all major muscle groups weekly [7,8]. Epidemiological evidence suggests that meeting both the aerobic and muscle-strengthening guidelines prospectively decreases all-cause mortality in adults since each type of activity has unique and cumulative benefits [9,10]. However, women are more likely to complete the aerobic portion of the guidelines. Over 50% of US adult women report meeting the aerobic guidelines only, while 24.3% meet the muscle-strengthening guidelines only [5]. Given that women are more likely to meet the aerobic but not the muscle-strengthening portion of the guidelines despite strong evidence that participating in both activities decreases all-cause mortality and improves quality of life, it is critical to examine women’s perceptions of and adherence to physical activity and exercise.

While physical activity and exercise are often used interchangeably, they are not the same [11,12]. Physical activity is any bodily movement produced by skeletal muscle that increases energy expenditure above resting metabolic levels [11,12]. Physical activity includes any active action or task due to daily life activities, active transportation, or leisure time physical activity [8]. Physical activities include but are not limited to walking to work, grocery shopping, and exercising. Exercise is a subset of physical activity that is planned, structured, and repetitive [11,12]. Exercise can be referred to as leisure-time physical activity since it is usually performed in an individual’s spare time. Therefore, it can be noted that increasing the amount of exercise a person does also increases their physical activity. Exercise includes sports, workout classes, recreational walking, running, and lifting weights [11]. Exercise is often categorized by domain. There are four primary exercise domains: (1) cardiorespiratory training, (2) resistance training, (3) neuromotor training, and (4) flexibility training [11]. To place exercise within the physical activity guidelines, aerobic exercise and cardiorespiratory training are often used interchangeably, while resistance training and muscle-strengthening activities are very similar [8]. For the sake of brevity, for the remainder of this manuscript, we will use physical activity as a blanket term to indicate physical activity, leisure-time physical activity, and exercise.

Research suggests that self-efficacy is closely related to empowerment [13,14,15,16], yet empowerment accounts for the influence of friends and family, the community, and even society [15]. Therefore, an empowered woman may participate in physical activity simply because she believes she can rather than to conform to societal standards, even when barriers arise [15]. Self-efficacy is the belief in one’s capabilities to organize and execute specific behaviors [17]. It is a key construct of empowerment theory, social cognitive theory, and the theory of planned behavior [18], and has been linked to enjoyment [19] as well as engagement in physical activity [18,20,21]. However, research suggests that exercise adoption is more successful when an activity is enjoyable but that self-efficacy is a driving factor for exercise adherence [19]. Nonetheless, self-efficacy is positively linked to the direction, intensity, and persistence with which actions are performed [22]. Along with enjoyment, it predicts physical activity initiation and maintenance over time [19].

Women’s reasons for exercise participation differ and could be related to self-efficacy and empowerment [23]. In fact, different types of physical activity have been identified as more empowering than others; thus, the mechanisms of empowerment likely vary [16,24,25,26,27]. For example, some argue that engagement in traditionally masculine activities, such as resistance training, team sports, or martial arts, empowers women to resist gender norms [16,24,27,28]. Women also describe increased empowerment from unique activities like pole dancing, roller derby, and powerlifting [27,29,30]. Overall, research suggests physical activities that shift focus from how the body looks to how it performs, such as resistance training [31], can be more empowering than others [30]. For women, resistance training engagement is an under-studied and promising avenue for further exploration [30]. Moreover, resistance training requires more skill development, and developing skills over time to participate in resistance training may increase self-efficacy [32]. 

Women participating in physical activity have increased physical competence, self-perception, and physical connectedness [25,26,27]. Studies that examine how these feelings relate to exercise empowerment are limited in part because a standardized measurement of exercise empowerment does not exist [33]. To our knowledge, no other study has asked women to rank physical activities and exercises they participate in from most to least empowering. Therefore, this study aims to describe which types of physical activities are ranked most empowering by women and explore the relationships between self-rated empowering exercise types, exercise engagement, and self-efficacy. We hypothesized that women would rank muscle-strengthening activities (e.g., resistance training) more empowering than aerobic activities (e.g., cardiorespiratory training). We also hypothesized that women empowered by muscle-strengthening activities would have more self-efficacy and, thus, do more physical activity. The secondary study purpose was to qualitatively understand why women rank certain types of physical activity as more or less empowering and to elucidate the relationship between empowerment and enjoyment in physical activity.

## 2. Materials and Methods

### 2.1. Study Design and Participants

An explanatory sequential mixed-methods study was conducted online and in person, where in-person interviews followed a cross-sectional survey. The cross-sectional survey was designed to explore how empowerment rankings varied between exercise types and to explore relationships between self-efficacy for exercise, physical activity engagement, and empowerment. The online survey allowed for a broader range of participants while minimizing participant burden. Survey participation was voluntary, and participants were not incentivized. Semi-structured interviews were conducted in person to help elucidate how different forms of physical activity foster empowerment. Participants who completed the interview were given a $20 gift card for their time.

Study participants were recruited using flyers displaying a QR code and a bit-link linked to the survey, distributed via email through the university’s daily newsletter and the Department of Kinesiology’s weekly newsletter. The flyer was also shared on social media platforms and by word of mouth. The survey was distributed online through Qualtrics (Qualtrics Labs Inc., Provo, UT, USA). Of the 306 people who clicked on the survey, 147 (48%) met the inclusion criteria and completed the survey entirely. To be eligible for the study, participants had to identify as female, be 18–65 years old, and live in the Midwest (i.e., Illinois, Indiana, Iowa, Kansas, Michigan, Missouri, Montana, Nebraska, North Dakota, Ohio, South Dakota, or Wisconsin, USA). The study received approval from the university’s Institutional Review Board, approval #IRB-11315, on 7 October 2022.

### 2.2. Survey Procedures

Upon participants meeting the inclusion criteria, the online survey described the study’s purpose and the confidentiality of responses before obtaining informed consent for the quantitative portion of the study, thus beginning the study. Following this, women entered basic demographic information, answered questions about their height and weight, completed the Godin–Shephard Leisure-Time Physical Activity Questionnaire (GSLTPAQ) and the Self-Efficacy for Exercise Questionnaire, and ranked up to five types of physical activity from most to least empowering (i.e., 1 = most empowering, 5 = least empowering). An optional open-ended question allowed participants to elaborate on their feelings of empowerment and physical activity. At the end of the survey, participants could enter their name and email in a separate secure URL if they were interested in completing an individual interview.

### 2.3. Survey Measurements

#### 2.3.1. Weight and Height

Participants self-reported their weight and height, as self-reported anthropometric measurements accurately calculate BMI for classification purposes [34]. Weight was reported in pounds as a whole number. Height was reported in feet and inches to the nearest quarter of an inch, then converted to a number in inches. Imperial measurements were retained for statistical analysis. 

#### 2.3.2. Body Mass Index (BMI)

BMI was calculated using standard procedures [35] where BMI = weight (lbs.)/[height (in.)] 2 × 703. BMI was categorized into four categories: (1) underweight (<18.5 kg/m^2^), (2) normal weight (18.5 to <25 kg/m^2^), (3) overweight (25.0 to <30 kg/m^2^), and (4) obese (≥30.0 kg/m^2^) [36].

#### 2.3.3. Godin–Shephard Leisure-Time Physical Activity Questionnaire (GSLTPAQ)

The GSLTPAQ is a short, widely used self-report measure commonly used to examine individuals’ current leisure-time physical activity levels. We asked participants, “During a typical 7-day period (one week), how many times on average do you do the following kinds of exercise for more than 15 min during your free time?” [37]. Their physical activity was classified as strenuous (heart beats rapidly), moderate (not exhausting), and light (minimal effort). The GSLTPAQ was scored according to standard procedures where exercise frequency was calculated from arbitrary units to metabolic equivalents. Participants were grouped into three categories: (1) insufficiently active (<14 units), (2) moderately active (14 to 23 units), and (3) active (≥24 units) [37]. 

#### 2.3.4. Self-Efficacy for Exercise Questionnaire

The nine-question Self-Efficacy for Exercise Questionnaire helps determine an individual’s self-efficacy to participate in physical activity despite facing nine common barriers [37]. This questionnaire is a valid and reliable measure (Cronbach’s α = 0.90) that asked participants: “How confident are you right now that you could exercise three times per week for 20 min if:” followed by nine different scenarios (e.g., they were experiencing pain during exercise, were not enjoying the exercise, they felt tired or stressed) [38]. Participants were asked to rate their confidence to still participate in physical activity when they faced each of the nine scenarios on a scale from 0–10, where 0 corresponded to not confident and 10 was very confident. The questionnaire was scored according to standard procedures [38]. Total scores could range from 0 to 90, with scores of 50 or higher indicating high self-efficacy for exercise.

#### 2.3.5. Open-Ended Empowerment Ranking Question

The open-ended empowerment ranking question allowed participants to enter up to five different leisure-time physical activities in which they currently or had participated. First, the Oxford Languages definition was provided, then participants ranked their physical activities by dragging and dropping each into a rank order from one to five. One indicated the most empowering activity, and five was the least empowering. The question was worded as follows:

According to Oxford Languages, the definition of empowerment is “the process of becoming stronger and more confident, especially in controlling one’s life and claiming one’s rights”.

List up to five leisure-time physical activities in which you participate(d). Rank these activities based on the level of empowerment you experience(d) by dragging and dropping them into the corresponding order: 1 being the most empowering activity and 5 being the least empowering activity. Examples of leisure-time physical activities include but are not limited to running, playing basketball, taking fitness classes, riding a bike, and lifting weights.

Two researchers (AS and ML) categorized each physical activity by primary exercise type as defined by the American College of Sports Medicine (i.e., cardiorespiratory training, resistance training, neuromotor training, and flexibility training) [39]. Given the wide variety in responses, this approach was utilized to make meaningful comparisons between groups. One researcher (ML) was the primary coder and categorized all 682 responses. The secondary coder (AS) analyzed 20% of the sample (*n* = 135). Coding agreement was very high (i.e., Cohen’s Κ = 94.5%). CrossFit^®^ was consistently categorized differently between the two researchers, but upon discussion, a consensus was reached, and CrossFit^®^ was re-categorized as neuromuscular training [10].

### 2.4. Individual Interview Procedures

A research team member contacted interested individuals to schedule an in-person interview at a convenient time for the participant. Interviews (n = 17) were held at the institution. All participants signed a separate informed consent before their interview began, each acknowledging that the interview would be recorded but that no identifying information would be retained. Two researchers conducted each interview using the same set of eight base and probing questions. Participants were asked to describe their leisure time physical activity habits and how participation made them feel, especially related to empowerment constructs and enjoyment. The questions are provided below:

1. Leisure-time physical activities are not essential activities of daily living (work, transportation) and include sports, exercise, and recreational walking. Tell me about your current involvement in leisure-time physical activity.
2. Explain why you engage in that type or those types of physical activity.
3. Do you prefer to participate in physical activity alone or with other people?
4. Please tell me how participating in physical activity makes you feel.
5. How does your participation in physical activity affect the rest of your day?
6. What keeps you motivated to participate in physical activity?
7. According to Perkins and Zimmerman [2], “Empowerment at the individual level is defined as a process by which individuals (1) perceive and gain control over personal issues, (2) understand their environment critically, and (3) take actions to influence the issues in their lives or communities”.
  - What do you think about this statement?
  - Explain whether or not PA is empowering for you.
  - What type of PA is most empowering for you?
8. How does physical activity enjoyment relate to empowerment?

Researchers met throughout the study’s interviews to assess whether data saturation occurred (i.e., no new themes emerged) [39]. After interviewing the 17 participants, the research team felt data saturation was achieved [40]. Each interview was transcribed verbatim and lasted approximately 30 min. Each transcription was sent to the corresponding participant for member checking to ensure accuracy and that the recorded responses resonated with their experiences. 

### 2.5. Statistical Analysis

An a priori power analysis was not completed for this study because no standardized physical activity empowerment measurement exists [33], though it could be used as a benchmark in the future. Data were analyzed in SPSS version 27 (IBM, Armonk, NY, USA). Means ± standard deviation (*M* ± *SD*) were calculated for all data. All data were screened for normality using the Shapiro–Wilk test. Independent sample *t*-tests were used to explore differences in BMI, physical activity engagement, and self-efficacy for exercise among women empowered by cardiorespiratory and resistance training, as sample sizes were too small among neuromotor and flexibility training-empowered women. 

### 2.6. Qualitative Analysis

Three members of the research team analyzed interview transcriptions using Dedoose version 9.0.17 (SocioCultural Research Consultants, LLC, Los Angeles, CA, USA). The primary researcher (AS) led coding efforts, while two secondary researchers (ML, HB) assisted. To some extent, all three researchers participated in the interview process, though the primary researcher participated in all interviews. An inductive, iterative thematic analysis was employed [41,42,43]. Thematic analysis is frequently used to help understand individual experiences in qualitative studies [41,42,43]. Each researcher independently coded the transcripts by highlighting words and phrases that could lead to understanding the study’s purpose. As new codes emerged, researchers routinely returned to the raw data (i.e., transcripts) analyzed earlier in the process and applied the relevant new codes. After every transcript had been coded, all three researchers met to ensure that individual code meaning was clear and duplicative codes were combined, resulting in 151 unique codes. These codes were organized into ten categories based on commonalities [43,44]. After forming categories, researchers returned to the literature to aid in theme development [43]. Four themes were developed that represented the ten categories. These themes were consistent with and built upon previous research. This process was iterative and involved moving back and forth between the raw data, codes, and categories multiple times to capture participants’ experiences accurately. Lastly, a negative case analysis was completed to check for conflicting viewpoints across the identified themes [45]. 

## 3. Results

Of the 147 participants who answered the survey completely, most respondents (86.4%) resided in Kansas, while 3.4% resided in Michigan and Missouri. Most respondents were white (91.2%), though six (4.1%) indicated being American Indian or Alaskan Native. Many participants had at least some college education (37.4%), while more had a bachelor’s degree or higher (44.2%). 

### 3.1. Survey Findings

Survey findings are summarized in Table 1. While on average, participants were overweight in terms of BMI, the largest percentage were normal weight, the sample was sufficiently active (i.e., scores were greater than 24 units) and had high self-efficacy for exercise (i.e., scores were greater than 50).

#### Open-Ended Empowerment Question Analysis

When categorized by exercise type, cardiorespiratory training included running, walking, swimming, rowing, etc.; resistance training included lifting weights, powerlifting, and weightlifting, etc.; neuromuscular training included CrossFit^®^; flexibility training included yoga and yardwork; and other activity included reading, etc. Descriptive statistics were coded by exercise type and are shown in Table 2. The highest-ranked empowering exercise was resistance training (*n* = 56, 38%), followed by running (*n* = 21, 14%), walking (*n* = 19, 12%), high-intensity interval training (*n* = 9, 6%), and both dance and hiking (*n* = 4 each, 3% each).

Independent *t*-tests were conducted to compare the cardiorespiratory and resistance training-empowered groups, as the sample sizes of the other groups were too small to make statistical comparisons. There were no significant differences between groups for any variables (i.e., for BMI *t*(136) = 1.13, *p* = 0.65, for total physical activity *t*(136) = 1.13, *p* = 0.11, for moderate-to-vigorous physical activity *t*(136) = 2.42, *p* = 0.06, and for self-efficacy for exercise *t*(136) = 0.66, *p* = 0.07). 

### 3.2. Individual Interview Findings

Thematic analysis of interviews (n = 17) resulted in four prominent themes, including (1) many barriers to female participation in physical activity are gender-centric, (2) the benefits of physical activity participation extend beyond physical health, (3) some types of exercise are more empowering than others, and (4) empowerment and enjoyment are closely related. Excerpts are quoted verbatim, except when brackets are used to provide clarity.

#### 3.2.1. Many Barriers to Women’s Participation in Physical Activity Are Gender-Centric

Most identified barriers to women’s participation in physical activity were directly related to being a woman. However, some, including a lack of knowledge and losing motivation, were cited by participants, as well. For example, one woman reported feeling frustrated due to her lack of knowledge about using the equipment. She said, “Actually, a reason I didn’t join a gym [was] because I wasn’t comfortable to go into a place where there’s just stuff, like no one leading you and [telling you] what to do.” Another remarked, “I used to go with my friend, but then she kind of got hurt, so she hasn’t been going as much, so I’ve been trying to keep going. Sometimes I’ve been losing the motivation to go since I’m by myself.” When probed, this participant stated that she did not like going to the gym alone because the environment was intimidating. Another participant echoed this feeling, “it is intimidating to walk into a gym, and you’re the only girl there.”

Analysis revealed that barriers to physical activity participation, especially at a gym, were mainly related to being a woman. Participants felt judged for being a woman, not lifting heavy, or being made to feel uncomfortable. One participant reported that working out in the gym is “super intimidating.” She said,

I’m a female, so a lot of times I felt [they] just stare[d] at me, or I’ve had people come up to me and go, “You’re not doing the right technique to lift weights.” And I’m like, “I’ve been lifting weights since I was 14. I know what I’m doing.”

One participant described her experience with the bench press exercise: “I’ve been doing a lot more benching recently, and that’s also nerve-wracking ‘cause I know I’m not lifting that much.” Another participant described her experience of feeling like she was not lifting enough weight, “I feel like I get a lot of eyes sometimes if I’m not lifting a lot of weight. Or I’m just doing like ten pounds or whatever.” She said she felt like a “little crab” in the corner of the gym, avoiding certain areas because they were male-dominant. Many participants felt the same way, noting certain areas they avoid. “Yeah, I would say I try to stay away from certain parts of the gym… OK, I can do it here ‘cause there aren’t going to be a lot of guys.” Several discussed male entitlement as a significant barrier to using specific machines in the gym. One said, “You’re not a male. It’s like, don’t expect to get the machines you want because you aren’t going to!" Still, another relayed an experience with a male gym-goer that escalated to stalking and required a restraining order. She said,

They reviewed footage and were like, “Yeah, he’s definitely following you. Like, we believe you.” So, that’s why I get so afraid when someone stares at me. I’m like, “I don’t like you staring at me because I’ve had [a] previous experience that was bad.” And so, I’m just like, please don’t stare at me. Please don’t. Don’t even come to me, don’t talk to me.

#### 3.2.2. The Benefits of Physical Activity Participation Extend beyond Physical Health

Participants could easily describe the physical, mental, and social health benefits of physical activity participation. For example, several participants discussed how physical activity made them feel strong, built their endurance, and was critical to maintaining health across the lifespan. Several participants stated that they “like feeling strong.” Others highlighted the benefits of building endurance, saying, “[Physical activity] allows me just to enjoy different outdoor activities and just not be winded.” Maintaining lifelong health was an essential benefit of physical activity among participants. One said, “As I grow older, I want to continue to have all the movements and like function that I have now,” while another echoed similar thoughts, saying, “I know long-term health is important,” and “I don’t want to age poorly and be at risk for more things.”

Mental health benefits included improvements in mood, confidence, and stress levels. One participant commented, “It always boosts my mood and helps clear my head, especially when I’m stressed.” Several participants conveyed similar experiences, “It’s a good mental break,” and “It just clears my head and boosts my mood.” Physical activity consistently contributed to an improvement in confidence among participants. One said, “I feel a lot more confident in myself and what I can do.” Another commented,

I struggled with body image issues when I was younger because probably because I wasn’t doing anything for myself. Like I was doing things like playing volleyball. That was for fun and not for my body[’s] health, but now that I participate in things that are good for my health and good for my body, I view my body in a more positive way.

Participants also cited the importance of physical activity to help destress and keep stress at bay. One said, “I feel like, if I had any stresses in the morning, [after working out] all of that’s like, gone.” Yet another commented, “I have a lot of stress from my classes right now, so I think [by] going to the gym, I can really escape my mind.” Still, another said, “[Physical activity] has been clinically proven to reduce stress, and lifting gives me a lot of destress time.”

#### 3.2.3. Some Types of Physical Activity Are More Empowering Than Others

Interview data corresponded with survey data in that most participants found lifting weights more empowering than participating in cardiorespiratory training. Central to feeling empowered were the feelings of accomplishment fostered by lifting weights. One participant said, “When I do strength training after[wards], it just makes you feel really strong, and like, I don’t know, I feel like I can conquer anything.” Similarly, another participant said,

After I do a strength training workout or something like that, and I feel, like, super strong, and like, I don’t know, I feel like I could really do just about anything after I finish that. And that makes me feel, like, very empowered.

Feeling accomplished fostered empowerment. One participant said, “I would say it’s it is empowering. Actually, I feel I like feeling stronger. I like getting to, like, lift heavier each time. It gives me a sense of accomplishment.” Similarly, another participant described how lifting heavier leads to feeling empowered. She said, “Like going up in weight, and being able to not ask for help, because I don’t want to ask for help ‘cause it’s all guys, so it’s, I’ve been very proud of myself.” It is important to note that feeling empowered by physical activity was not limited to resistance training. 

Cardiorespiratory training activities like running were empowering to some participants, but again, feeling a sense of accomplishment was critical to feeling empowered. For example, one participant said, “My favorite feeling ever is when you get towards the end of a race, and you’re dead tired, but like, you get a sudden burst of energy and feel like you can just keep going forever.” However, activities like walking did not elicit the same feelings of empowerment. One participant commented,

I think like walking or stuff like that is enjoyable, and like, it’s fun to do with someone, but I don’t get done with that, and I’m like, “Man, I just accomplished so much,” and like, I’m not super empowered by that type of activity.

Others felt similarly, “Even if I [did] choose to go on a walk for like exercise, I won’t say that’s super empowering ‘cause it doesn’t feel difficult,” and “Leisurely walking isn’t, like, super empowering because a lot of people can do it, but it’s still enjoyable.”

#### 3.2.4. Empowerment and Enjoyment Are Closely Related

Empowerment was described as a multidimensional construct that affected participants at the individual, interpersonal, and community levels and was closely related to physical activity enjoyment. At the individual level, many participants expressed how setting aside time to exercise was something they did to take care of themselves. For example, one woman said, “I do it [resistance training] kind of for myself, I guess. It makes me feel good.” Another commented,

Being physically active gives me time to, like, recognize things I want to, like, change about myself or, like, my environment. It’s like a place for the growth for me basically, and it allows me to like think deeply on those ‘cause that’s like my thinking time. I’m walking, and I’m like, hmm, I gotta do this, and I want to do this, so I think it gives me time just to do a lot of, like, thinking about different things going on my life and how to handle those.

When talking about lifting weights, one participant commented, “I’m doing something to make my life better, which, like, in turn, makes everything else kind of better.” At the interpersonal level, participants discussed how exercising with other women friends could be empowering. For example, one participant said, “I feel a lot more confident in myself and what I can do. As well as, like, how I can, like, push myself to reach my goals ‘cause I started going with friends to the gym to lift.” On the other hand, some participants noted that the type of activity mattered. Several described enjoying time with friends but not feeling empowered by the physical activity type. “I enjoy the community aspect of hanging out with friends and playing whatever sport, but it doesn’t...You don’t leave being like, yes, I accomplished something.” Feeling a sense of community also contributed to enjoyment and empowerment. One participant commented,

I think the community aspect was really important to the gym I belonged in, and that definitely made me more comfortable working out. And now when I’m around certain people, I feel like there is a community in the gym, and sometimes people don’t really talk to each other, but today a lot of people were, like, talking and communicating, and I was like, this is weird, but it was kind of nice because everyone was, like, sharing equipment.

Enjoyment was critical to feeling empowered. One woman said, “The more you love something and the more you’re enjoying it, the more empowered you’re going to feel at the end of it. At least for me.” Another echoed this sentiment, stating, “So I think with me enjoying the type of physical activity I do, it really helps me feel more empowered because if I’m not going to enjoy it, then I’m not going to do it.”

## 4. Discussion

### 4.1. Overall Discussion

This study examined how women ranked different physical activities and exercises as empowering, their self-reported engagement in physical activity, and their self-efficacy for exercise. The main findings reveal that the largest group of women ranked resistance training as the most empowering form of physical activity in which they participate. This finding supports the study’s hypothesis that muscle-strengthening exercises like resistance training are more empowering than cardiorespiratory training. While resistance training-empowered women, on average, participated in more total and moderate-to-vigorous physical activity and had higher average self-efficacy for exercise than cardiorespiratory-empowered women, these differences were not statistically significant. Therefore, our second hypothesis was not supported as there were no statistically significant differences in physical activity participation or self-efficacy for exercise between resistance training-empowered women and cardiorespiratory-empowered women.

Furthermore, our qualitative examination of why women rank different activities as more or less empowering supported the multidimensional construction of empowerment theory as women described the contribution of the individual, interpersonal, and community levels [2,3]. At the individual level, they noted that physical activity positively affected their physical, mental, and social health. They felt empowered to do physical activity because they knew how beneficial it was. The women in our study discussed how working out with friends related to the interpersonal level. Some women noted that exercising with others held them back, especially during cardiorespiratory activities like walking and running. At the community level, participants discussed the importance of women role models and noted that male gym-goers were sometimes a barrier to doing resistance training. While some commented on being in the zone and doing their own thing, they acknowledged that seeing the same people at the gym contributed to a sense of community. While all three levels of empowerment were represented in our data, almost all participants described how feeling a sense of accomplishment was critical to feeling empowered. They remarked that feeling accomplished and thus empowered was fostered by resistance training more than other physical activities. Additionally, participants indicated that physical activity must be enjoyable to be empowering, but for some, activities like walking were enjoyable but not empowering. 

Our results highlight how physical activity can be empowering. A small but growing body of literature aligns with this result [21,22]. For example, one community-based participatory research study among disadvantaged women focused on empowerment outcomes [21]. Researchers found that their program enhanced participants’ empowerment by increasing their self-efficacy, competency development, and motivation to do physical activity [21]. Another study evaluated a six-month empowerment-based exercise intervention program among sedentary Swedish girls [22]. Girls who completed the intervention learned skills to help them effectively manage stressful situations and improve their physical fitness [22]. Unsurprisingly, empowerment-based physical activity interventions positively impacted participants through various mechanisms, including increased self-efficacy [21,22].

The quantitative and qualitative portions of our results support the notion that specific types of physical activity are more empowering than others. Among women in our study, resistance training was ranked as the most empowering form of physical activity in which they participated in the quantitative portion of our study. Resistance training was also qualitatively discussed as more empowering than other forms of physical activity in the study’s interviews. Interview data help clarify why the women in our study found resistance training empowering. We learned that feeling a sense of accomplishment is critical to feeling empowered. Resistance training fostered a sense of accomplishment rooted in feeling and looking strong. Notably, running faster or farther was empowering for some of our participants. These results align with previous research in some ways. Historically, resistance training has been classified as a male-dominant activity [46]. Previous research indicates that women who engage in traditionally masculine activities like resistance training are empowered to resist gender norms [16,24,27,28]. However, while the women in our study described gender-based barriers to resistance training, they did not expressly state that they did resistance training to buck gender norms. Instead, feeling and looking strong was associated with feeling empowered. Similarly, one study that examined women’s experiences found that resistance training contributed to the development of self-efficacy, positive body image, and psychological well-being [30]. In addition, women described activities that contributed to a sense of accomplishment as more empowering than easy activities. Some of the most popular physical activity interventions for women rely upon easy activities like walking [47]. However, our results suggest that activities that cultivate a sense of accomplishment may increase self-efficacy and engagement in moderate-to-vigorous physical activity.

Self-efficacy is an essential facet of empowerment and physical activity engagement. To our knowledge, this is the first study that examined how empowerment from different types of physical activity contributes to self-efficacy and engagement in exercise and physical activity. On average, study participants scored above average self-efficacy for exercise. Resistance and neuromotor training-empowered women scored over 50 (i.e., the threshold for high exercise self-efficacy). These findings may be partly due to the skill specialization required for these types of exercises [32]. Yet, cardiorespiratory-empowered women also scored high on average (i.e., 48.5) on the self-efficacy for exercise scale. Therefore, it is worth considering that individuals who respond to a survey about exercise and empowerment are more likely to have knowledgeable about and high self-efficacy for exercise.

Our findings align with previous research that describes the positive relationship between self-efficacy and physical activity initiation and maintenance among women. For example, research suggests that improvements in physical activity are mediated by self-efficacy among working mothers who engage in a brief physical activity intervention [20]. Self-efficacy improved among disadvantaged women and Swedish girls who participated in empowerment-based interventions [21,22]. The relationship between self-efficacy and empowerment seems to be bidirectional, where improvements in self-efficacy led to feeling empowered and vice versa. Self-efficacy facilitates the initiation and maintenance of physical activity and is closely related to empowerment.

### 4.2. Suggestions for Practice

Since empowerment accounts for the individual, their friends and family, and the community in which they live, all three levels must be targeted to increase women’s empowerment through physical activity. In particular, teaching women how to do resistance training may be impactful. Creating supportive groups of women who lift will also increase women’s resistance training participation. Lastly, inclusive gym spaces are required so women feel comfortable doing resistance training and other physical activities. Given the well-documented physical, mental, and social benefits of physical activity, it is time for a renaissance that promotes all types of physical activity for women, especially resistance training.

### 4.3. Strengths and Limitations

Study strengths include implementing a sequential mixed-methods design where survey data were explored in greater depth through 17 semi-structured qualitative interviews. The survey used two validated and reliable questionnaires to assess physical activity engagement and self-efficacy for exercise. The open-ended empowerment ranking question allowed participants to freely type in any physical activity or exercise they participated in; thus, they were not limited to certain types of physical activity. Two researchers classified all physical activity into five categories based on training adaptation and had a very high (i.e., 94.5%) agreement. Our iterative and inductive approach to analyzing the interview data was considered valid and reliable, and we achieved data saturation through our interviews.

However, several study limitations should be addressed. Our study sample is not representative as it was predominantly white and well-educated women. As with any cross-sectional design, the data we captured from our survey are simply a snapshot of participants’ experiences and preclude causality. Some participants were more active than others, and we did not measure the type of physical activity they participated in, only the intensity. Therefore, it is possible, for example, that someone who ranked resistance training as the most empowering form of physical activity they participated in was doing low-intensity physical activity like walking. Longitudinal studies may better capture the empowerment process through physical activity and better elucidate how physical activity type impacts feelings of empowerment, self-efficacy, and adherence to physical activity. Moreover, differences in total and moderate-to-vigorous physical activity and self-efficacy for exercise were not statistically significant between cardiorespiratory- and resistance training-empowered women, which may indicate that our study was not adequately powered. Future research should include a larger, more diverse sample and account for cultural and religious barriers among minority groups.

## 5. Conclusions

This explanatory sequential mixed-methods study adds insight into which types of physical activities women see as empowering. The women in our study ranked resistance training as more empowering than other physical activity types. This is notable since epidemiological evidence suggests women are less likely than men to do muscle-strengthening activities like resistance training. Running, walking, HIIT, dancing, and hiking were among the top five most empowering physical activities identified as empowering by women in our study. We also found that women understand the benefits of physical activity but face gender-specific barriers like feeling judged by male gym-goers or not knowing how to use the equipment. In our study, feeling accomplished after a workout was related to feeling empowered. Many women described how being able to do hard things like lifting heavy weights or running long distances was empowering.

## Figures and Tables

**Table 1:** Survey participant characteristics (*N* = 147).

Variable	*M* ± (*SD*)	*N* (%)
Height (in)	65.5 ± (2.7)	
Weight (lbs.)	156.2 ± (35.7)	
Body Mass Index (kg/m^2^)	25.1 ± (6.6)	
Underweight (<18.5 kg/m^2^)		3 (2.0)
Normal Weight (18.5–24.9 kg/m^2^)		77 (53.5)
Overweight (25–29.9 kg/m^2^)		38 (26.4)
Obese (≥30 kg/m^2^)		26 (18.1)
Missing		3 (2.0)
Total Physical Activity	53.5 ± (27.2)	
Moderate-to-Vigorous Physical Activity	40.1 ± (23.9)	
Self-Efficacy for Exercise	50.7 ± (19.6)	

**Table 2:** Descriptive statistics for categories of most empowering activity by exercise type (presented as *M* ± *SD*).

Variable	Cardiorespiratory Training-Empowered (*n* = 81)	Resistance Training-Empowered (*n* = 57)	Neuromotor Training-Empowered (*n* = 3)	Flexibility Training-Empowered (*n* = 3)	Other Activity-Empowered (*n* = 3)
BMI	25.2 ± (7.2)	24.7 ± (6.0)	24.9 ± (2.1)	26.5 ± (1.3)	29.0 ± (9.3)
Total Physical Activity	51.7 ± (27.0)	59.2 ± (25.9)	53.3 ± (31.9)	24.7 ± (16.2)	14.3 ± (9.2)
Moderate-to- Vigorous Physical Activity	37.9 ± (25.4)	45.5 ± (21.3)	49.3 ± (31.3)	17.7 ± (15.5)	8.3± (14.4)
Self-Efficacy for Exercise	48.5 ± (20.7)	54.8 ± (18.2)	52.7± (7.6)	33.7 ± (8.0)	48.7 ± (18.1)

## Data Availability

Data are available upon request from the corresponding author.
